# Natural neopolyploids: a stimulus for novel research

**DOI:** 10.1111/nph.20437

**Published:** 2025-02-14

**Authors:** Patrick P. Edger, Douglas E. Soltis, Shunsuke Yoshioka, Mario Vallejo‐Marin, Rie Shimizu‐Inatsugi, Kentaro K. Shimizu, Armel Salmon, Simon Hiscock, Malika Ainouche, Pamela S. Soltis

**Affiliations:** ^1^ Department of Horticulture Michigan State University East Lansing MI 48823 USA; ^2^ Genetics and Genome Sciences Michigan State University East Lansing MI 48824 USA; ^3^ Florida Museum of Natural History University of Florida Gainesville FL 32611 USA; ^4^ Department of Biology University of Florida Gainesville FL 32611 USA; ^5^ Department of Evolutionary Biology and Environmental Studies University of Zurich Zurich CH‐8057 Switzerland; ^6^ Graduate School of Agriculture Kyoto University Kyoto 244‐0813 Japan; ^7^ Department of Ecology and Genetics, Evolutionary Biology Centre Uppsala University Uppsala 752 36 Sweden; ^8^ Kihara Institute for Biological Research Yokohama City University Yokohama 641‐12 Japan; ^9^ UMR CNRS Ecobio Rennes University Rennes Cedex 35042 France; ^10^ Department of Biology University of Oxford Oxford OX1 3RB UK

**Keywords:** allopolyploid, angiosperms, genetic and phenotypic variation, genome evolution, neopolyploidy, polyploidy, whole‐genome duplication

## Abstract

Recently formed allopolyploid species offer unprecedented insights into the early stages of polyploid evolution. This review examines seven well‐studied neopolyploids (we use ‘neopolyploid’ to refer to very recently formed polyploids, i.e. during the past 300 years), spanning different angiosperm families, exploring commonalities and differences in their evolutionary trajectories. Each neopolyploid provides a unique case study, demonstrating both shared patterns, such as rapid genomic and phenotypic changes, and unique responses to hybridization and genome doubling. While previous studies of these neopolyploids have improved our understanding of polyploidy, significant knowledge gaps remain, highlighting the need for further research into the varied impacts of whole‐genome duplication on gene expression, epigenetic modifications, and ecological interactions. Notably, all of these neopolyploids have spontaneously arisen due to human activity in natural environments, underscoring the profound consequences of polyploidization in a rapidly changing world. Understanding the immediate effects of polyploidy is crucial not only for evolutionary biology but also for applied practices, as polyploidy can lead to novel traits, as well as stress tolerance and increased crop yields. Future research directions include investigating the genetic and epigenetic mechanisms underlying polyploid evolution, as well as exploring the potential of neopolyploids for crop improvement and environmental adaptation.

**Table:** 

	Contents	
	Summary	78
I.	Introduction	79
II.	Genetic and epigenetic changes following polyploidization	81
III.	Role of hybridization vs genome doubling	83
IV.	Genotypic and phenotypic diversity	84
V.	Reproduction	85
VI.	Repeated evolutionary experiments	86
VII.	New polyploids afford unique opportunities to distinguish inherited traits and rapid evolution	87
VIII.	Changes through time – newly formed vs older polyploids	87
IX.	Every polyploid tells a story	88
X.	Factors of formation	88
XI.	Major gaps: other facets of evolution to be learned from new polyploids	88
XII.	Conclusions	89
	Acknowledgements	90
	References	90

## I. Introduction

**Fig. 1:**
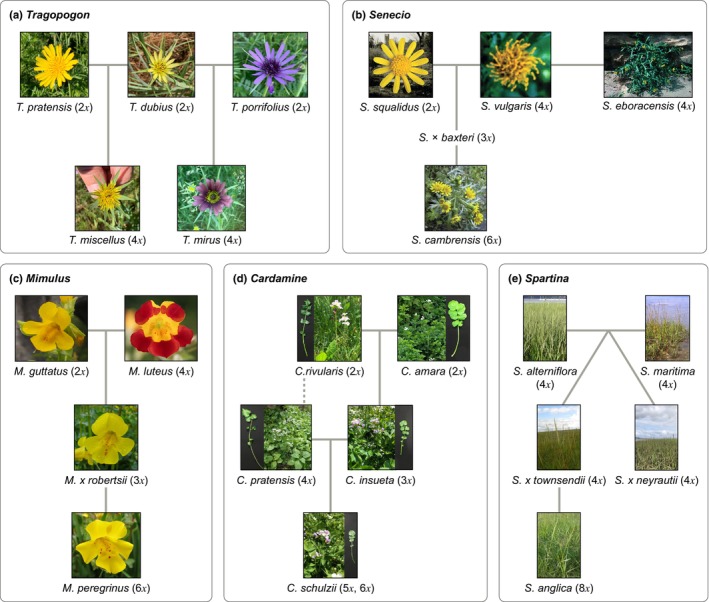
Evolutionary history of five polyploid complexes. (a) *Tragopogon* polyploid complex. The tetraploid species *T. miscellus* and *T. mirus* arose from hybridization and polyploidization of their respective diploid parental species: *T. pratensis* and *T. dubius* for *T. miscellus*, and *T. dubius* and *T. porrifolius* for *T. mirus*. (b) *Senecio* polyploid complex. The hybridization of the diploid species, *S. squalidus*, and tetraploid species, *S. vulgaris*, created the triploid intermediate species, *S. × baxteri*, and following a polyploidization event created the hexaploid species, *S. cambrensis*. The hybridization of *S. vulgaris* and *S. squalidus* produced an F_1_ hybrid and following subsequent backcrossing of this F_1_ hybrid with *S. vulgaris* led to the formation of *S. eboracensis*. (c) *Mimulus* polyploid complex. The hybridization of the diploid species, *M. guttatus*, and tetraploid species, *M. luteus*, created the triploid intermediate species, *M. × robertsii*, and following a polyploidization event created the hexaploid species, *M. peregrinus*. (d) *Cardamine* polyploid complex. The hybridization of the diploid species, *C. rivularis* and *C. amara*, created the triploid species, *C. insueta*, and following hybridization with the autotetraploid species, *C. pratensis*, created the mixed ploidy (5*x* and 6*x*) species, *C. schulzii*. The autotetraploid species *C. pratensis* arose from the polyploidization of a lineage close to *C. rivularis*. (e) *Spartina* polyploid complex. Two tetraploid species, *S. alterniflora* and *S. maritima*, hybridized to form two interspecific homoploid hybrids, *S. × townsendii* and *S. × neyrautii*. The octoploid species *S. anglica* arose following the polyploidization of the tetraploid *S. × townsendii*.

Polyploidy occurs frequently across the tree of life, not only in eukaryotes but also in Bacteria and Archaea (reviewed in Morris *et al*., 2024), and has played a particularly important role in the evolution of green life (Heslop‐Harrison *et al*., 2023). Hence, long‐standing research has attempted to identify the diverse consequences of polyploidy in plants at multiple levels (e.g. genetic, genomic, physiological, morphological, and ecological) and the role of polyploidy in evolutionary innovation (Levin, 1983; Soltis *et al*., 2015; Van de Peer *et al*., 2017, 2021). Most polyploid species and lineages studied to date formed thousands to millions of years ago, making it challenging to disentangle the immediate impact of genome doubling from longer‐term evolutionary processes (Soltis *et al*., 2016; Freeling, 2017). However, a few studies have focused on naturally occurring polyploids that formed more recently, in the past 300 years (i.e. neopolyploids as defined here): *Cardamine insueta* and *C. schulzii* (Brassicaceae), *Mimulus peregrinus* (Phrymaceae), *Spartina anglica* (Poaceae), *Senecio cambrensis* (Asteraceae), and *Tragopogon mirus* and *T. miscellus* (Asteraceae; Fig. 1). These species have served as model organisms, affording the novel opportunity to investigate the earliest stages of polyploid evolution in nature, something that can be simulated in other species only via the use of synthetic polyploids or via quickly reproducing plants in laboratory culture (Bafort *et al*., 2023).

Neopolyploids, due to their recent origin, provide a unique opportunity to study the immediate effects of polyploidization, allowing researchers to separate the impact of genome doubling from subsequent evolutionary changes. Unlike older polyploids, where extended periods of evolution have led to the divergence of the polyploid from its progenitor(s), neopolyploids offer a snapshot of the early stages of polyploid evolution. This allows identification of key genetic and epigenetic changes that happen immediately after polyploidization, such as gene loss, silencing, and transposon activation, which may be critical for polyploid establishment and adaptation.

Studying natural neopolyploids offers significant advantages over the study of older, established polyploids and synthetic lines. We can observe the immediate impact of interspecific hybridization and genome doubling on factors, such as epigenetic marks, gene expression, and meiotic chromosome pairing. These initial changes are often stabilized in older polyploids, hindering examination of the immediate changes occurring following polyploidy in natural conditions. Neopolyploids allow direct comparison with the exact (or very close) parental genotypes from which they arose, an advantage not possible with older polyploids where both parents and polyploids have evolved and parental species might be extinct. In some polyploid systems, naturally established neoallopolyploids can be directly compared with not only their parents but also resynthesized hybrids and allopolyploids. This allows for deeper insights into the earliest epigenetic and genetic changes that occur in polyploid genomes (Edger *et al*., 2017). Neopolyploids also provide insights into early niche occupation and ecological interactions with parental species. Finally, the recurrent formation of the same polyploid allows researchers to assess parallel evolution and determine whether evolution repeats itself – that is, are some aspects of polyploid evolution actually hard‐wired?

Despite these advantages, challenges remain. The evolutionary trajectories of neopolyploids are uncertain. The short evolutionary timescale limits the observable changes, hindering a full assessment of the long‐term success of polyploid lineages. However, the benefits of studying neopolyploids outweigh these limitations. They offer invaluable insights into the initial stages of polyploid evolution, which are crucial for understanding the broader role of polyploidy in shaping biodiversity and driving agricultural innovation. Research on these species should be coordinated and continued by the next generation of plant scientists.

Here, we review what is collectively known about seven recently formed and well‐studied naturally occurring neoallopolyploids (Fig. 1) and attempt synthesis and identification of broader implications and future research directions. We assess whether there are rules to the early stages of polyploidy. However, we acknowledge the inherent limitations in drawing unified conclusions across diverse model systems, given the variation in research objectives and methodologies. One goal of this review was to stimulate more coordinated/unified research on these systems in the future. The studies discussed herein were selected to represent a breadth of polyploid systems, with a focus on (epi)genetic and phenotypic changes that have been documented in neopolyploids. Specifically, this review focuses solely on recently established allopolyploids and does not cover neoautopolyploids. Our approach, while not exhaustive, aimed to highlight key trends and knowledge gaps in our understanding of allopolyploid evolution. Each section below reviews the current state of knowledge and concludes with questions for future study, with the goal of stimulating new research on recent polyploidy.

Some of the polyploid systems reviewed here have reformed repeatedly, providing the opportunity to assess whether evolution repeats itself. Although commonalities are present across these polyploids, each new polyploid also represents a unique storyline and opportunity to investigate the impact of genome doubling. Although much has been learned about polyploid evolution from these newly formed species, major gaps in our understanding remain. In many cases, differing aspects of polyploidy have been studied (Madlung, 2013; Soltis *et al*., 2016). These new polyploids have all formed via human‐mediated activity, suggesting major impacts of polyploidization for a planet undergoing rapid global change. Lastly, understanding the impacts of polyploidy on recent timescales has major implications not only for evolutionary biology but also for agricultural research.

## II. Genetic and epigenetic changes following polyploidization

### 1. The search for ‘rules’ via the study of young polyploids

**Fig. 2:**
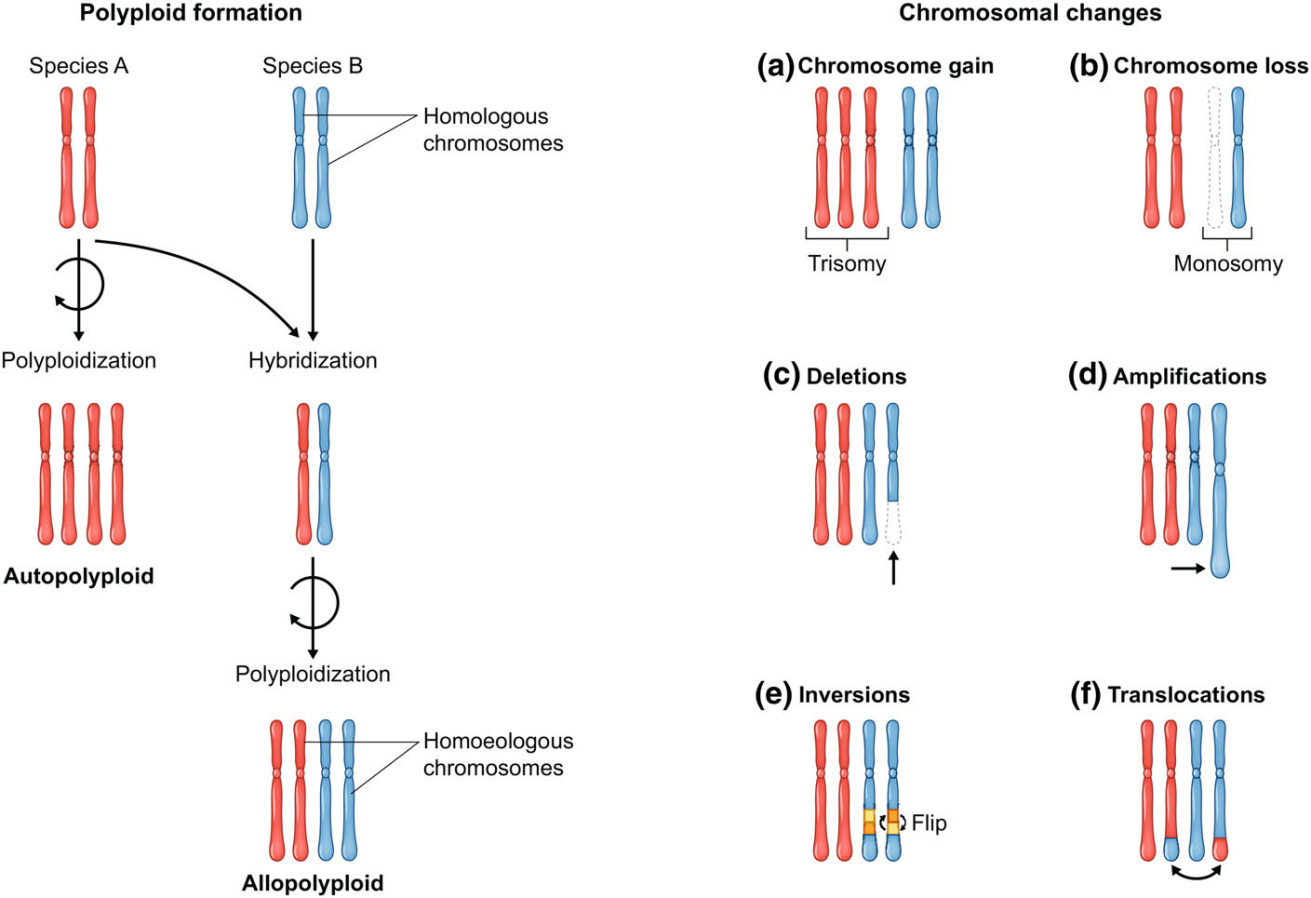
Polyploidization and its impact on chromosomal evolution and polyploid formation. Polyploid organisms possess more than two complete sets of chromosomes. This phenomenon can arise through autopolyploidy, where the additional chromosomes are derived from the same species, typically due to errors during cell division. Alternatively, allopolyploidy involves the combination of chromosome sets from different species through hybridization and genome duplication. It is important to note that an allopolyploid could be formed by the hybridization of two autopolyploids (i.e. polyploidy preceding hybridization) or via an unreduced gamete and/or triploid bridge (Vallejo‐Marín *et al*., 2015). Homologous chromosomes are pairs of chromosomes that share the same genes in the same order, inherited from each parent in diploid organisms, while homoeologous chromosomes share a common ancestry and are similar but not identical to chromosomes derived from different ancestral species in allopolyploid organisms. Polyploidization sets the stage for dynamic chromosomal evolution. Newly formed polyploids can experience gain (a) or loss (b) of entire chromosomes or segments thereof, affecting gene dosage and expression. Partial chromosome deletions (c) may remove crucial genes or regulatory elements, while amplifications (d) create additional copies with potential functional consequences. Chromosomal rearrangements such as inversions (e) and translocations (f) shuffle gene order and create novel combinations, contributing to the genetic and phenotypic diversity seen in polyploids.

Polyploid organisms arise through two main processes, which represent ends of a continuum: autopolyploidy, where duplicated chromosomes are derived from within the same species; and allopolyploidy, our focus here, where sets of chromosomes are derived from two or more species (Fig. 2). The process of polyploidization often leads to extensive genomic changes, including duplications, deletions, inversions, and translocations (Soltis *et al*., 1993; Han *et al*., 2003; Lim *et al*., 2008; Mandáková *et al*., 2010) and significant changes in gene expression, so‐called ‘transcriptome shock’ (Hegarty *et al*., 2006; Parisod *et al*., 2009; Chelaifa *et al*., 2010; Buggs *et al*., 2011; Hegarty *et al*., 2011), which can be accompanied by detectable epigenetic changes (Parisod *et al*., 2009; Buggs *et al*., 2011; Hegarty *et al*., 2011).

Genomic changes eventually lead to alterations in base chromosome number and structure over deep evolutionary time (Murat *et al*., 2015; Lysak *et al*., 2016) but can sometimes be observed in the earliest generations in some polyploids (Xiong *et al*., 2011; Chester *et al*., 2012; Soltis *et al*., 2016). This restructuring can result in alterations in gene dosage, where the expression levels of genes may be altered due to changes in absolute copy number and the relative number of parental subgenome copies (Fig. 2). Consequently, genes involved in various cellular processes, such as growth, development, and reproduction, may exhibit altered expression patterns in polyploids compared with their diploid progenitors (Birchler & Veitia, 2012; Jeffrey Chen & Birchler, 2013). Changes in relative gene dosage of parental copies have been shown to contribute to rapid novel phenotypic variation. For example, variation in flowering time, beyond the extremes observed for either diploid progenitor, was observed across a newly formed allotetraploid rapeseed (*Brassica napus*) population and was correlated with relative dosage of parental alleles of *Flowering Locus C* genes (Pires *et al*., 2004). Beyond changes in gene dosage, duplicated genes will accumulate mutations over time, leading to functional diversification and/or loss of function (Ohno, 1970; Force *et al*., 1999; Maere *et al*., 2005). The redundancy provided by multiple gene copies can facilitate the evolution of novel or diversified gene functions (Ohno, 1970). This can occur through neofunctionalization, where one duplicate acquires a new function, or subfunctionalization, where the ancestral gene's functions are partitioned between the duplicates. Both processes are driven by changes to regulatory and/or coding sequences (Lynch & Conery, 2000).

Gene expression changes in polyploids have been reported in multiple studies (Stupar *et al*., 2007; Grover *et al*., 2012; Fasano *et al*., 2016; Visger *et al*., 2019). In the recently formed allopolyploid *Senecio cambrensis*, massive changes in gene expression (termed ‘transcriptome shock’) were observed in first‐generation synthetic hybrids (which are sterile) compared with their parents and with subsequent generations of allopolyploids (which are fertile) after colchicine‐induced chromosome doubling (Hegarty *et al*., 2005, 2006, 2008). Similarly, immediate changes in gene expression were observed in naturally occurring and synthetic *Tragopogon mirus* and *T. miscellus* (Buggs *et al*., 2009, 2011, 2012). Significant transcriptome repatterning has occurred following recent allopolyploidy in *Spartina*, where expression changes (consistent with epigenetic and regulatory alterations) that took place in 150–170‐year‐old hybrids and neoallopolyploid *S. anglica* far exceeded long‐term divergent transcriptome evolution in the parents, which themselves are meso‐polyploids (Giraud *et al*., 2021).

**Fig. 3:**
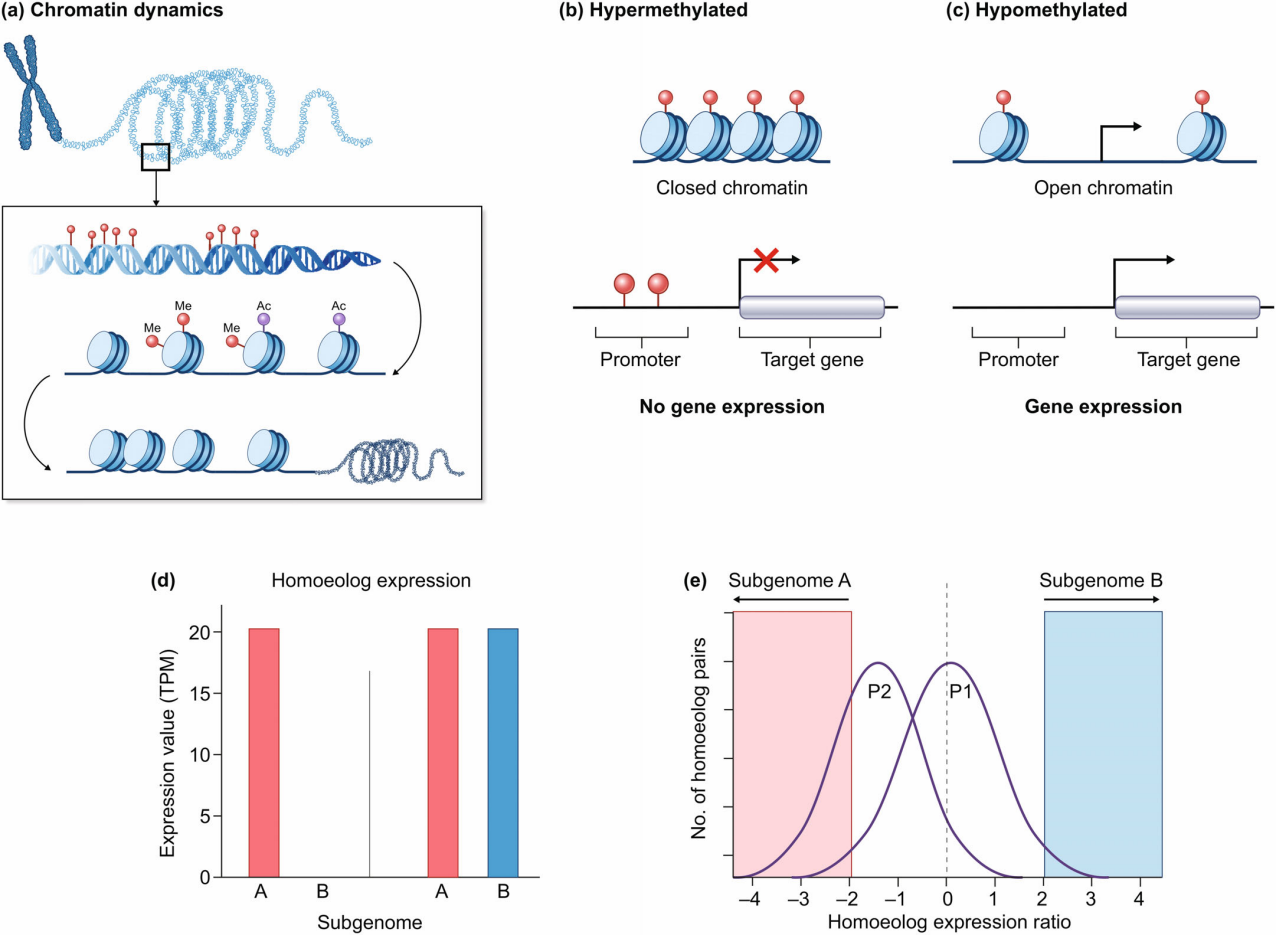
Polyploidy‐induced epigenetic and transcriptome changes. Polyploidization may trigger substantial epigenetic changes, influencing gene expression and shaping the new polyploid's phenotype. (a) Chromatin, the complex of DNA and proteins that packages the genome, undergoes dynamic remodeling in response to polyploidization. (b) DNA methylation, a key epigenetic mark, can be altered, leading to hypermethylation (increased methylation) or (c) hypomethylation (decreased methylation) of specific genomic regions. These changes influence chromatin structure, with hypermethylation typically associated with condensed, closed chromatin and hypomethylation with open chromatin. In the promoter region of genes, DNA methylation changes directly impact the accessibility of transcription factors, thereby regulating gene expression levels. (d) Subgenome expression dominance is observed in some allopolyploids, where one of the parental subgenomes exhibits higher overall gene expression levels compared with the other subgenome, as seen in the left panel, compared with equal expression in the right panel. (e) Homoeolog expression ratio refers to the relative expression levels of homoeologous gene pairs, which originate from different parental subgenomes (A and B) in allopolyploids. In one polyploid (P1), the overall homoeolog expression ratio shows balanced expression, indicating that neither subgenome exhibits significant dominance. However, in the other polyploid (P2), gene expression levels are heavily biased toward subgenome A. Ac, acetylation; Me, methylation.

In addition to genomic and transcriptomic changes, polyploidy can profoundly influence epigenetic regulation, the heritable modifications to DNA and chromatin that affect gene expression without altering the underlying DNA sequence (Fig. 3). Epigenetic mechanisms, including DNA methylation, histone modifications, and small RNA‐mediated silencing, play crucial roles in shaping the transcriptome, and ultimately the phenotypic plasticity, of organisms (reviewed in Osborn *et al*., 2003; Zhang *et al*., 2018). Hybridization, a key component of allopolyploid formation, can lead to immediate genome‐wide alterations in DNA methylation patterns (Madlung *et al*., 2002; Edger *et al*., 2017), often associated with the activation of transposons (‘genomic shock’ *sensu*; McClintock, 1984; de Tomás & Vicient, 2023). Hypermethylation and hypomethylation events can occur at specific genomic loci, affecting gene expression and contributing to phenotypic variation among polyploid individuals (Song & Chen, 2015; Mattei *et al*., 2022). Furthermore, changes in histone modifications, such as acetylation, methylation, and phosphorylation, can influence chromatin structure and accessibility, thereby regulating gene expression (Zhang *et al*., 2018). In the recent allopolyploid *T. miscellus*, methylation patterns were largely additive of the parents, although some novel differentially methylated regions were observed, again illustrating the ability of polyploidy to generate novelty (Shan *et al*., 2024).

Small RNAs, including microRNAs and small interfering RNAs, also contribute to the epigenetic regulation of gene expression (Holoch & Moazed, 2015; Cecere, 2021) and may be altered following allopolyploidy (Cavé‐Radet *et al*., 2020, 2023). These small RNAs can target specific mRNAs for degradation or translational repression, thereby fine‐tuning gene expression and buffering against potential dosage imbalances resulting from polyploidization (Birchler & Veitia, 2012; Conant *et al*., 2014). However, while the impact of polyploidy on mRNAs has been studied in various ways across multiple species, how other types of RNAs, including long noncoding RNAs (Palos *et al*., 2023), evolve following polyploid events remains poorly understood (Mohammadin *et al*., 2015; Chekanova, 2015). Similarly, beyond a few studies investigating DNA methylation changes in synthetic or naturally established polyploids, most other epigenetic markers, which may contribute to subgenome differences in accessible chromatin (Fang *et al*., 2024), have not been thoroughly studied nor in a holistic framework. Overall, the genetic and epigenetic changes that occur in polyploids are complex and multifaceted, reflecting the dynamic interplay between genomic and epigenomic processes. Understanding these changes is crucial for unraveling the evolutionary significance of polyploidy and its implications for biodiversity, adaptation, and speciation.

- There appear to be some ‘rules’ to polyploidy, including immediate regulatory/expression changes and instantaneously (or nearly so) inherited traits; these changes may affect the long‐term evolutionary fate of polyploid genomes (i.e. subgenome dominance, fractionation – the loss of duplicate gene copies – and concomitant genome size reduction).
- Many questions remain:
  ○ How does a newly formed cell in an allopolyploid control divergent (epi)genetic programs?
  ○ Do parental subgenomes exhibit distinct spatial organization within the nucleus, occupying different nuclear domains?
  ○ Which (epi)genetic changes contribute to specific observed phenotypic changes?
  ○ What proportion of subfunctionalization among retained duplicated genes is attributable to changes in regulatory sequences vs changes in protein‐coding sequences?

## III. Role of hybridization vs genome doubling

### 1. Assessing the relative impacts of hybridization and genome duplication

Allopolyploid speciation involves two distinct processes: the merger of divergent genomes (resulting from hybridization); and genome duplication. These processes may have different consequences that may be detected at both the phenotypic and the genetic/genomic levels. Neopolyploids provide critical information for disentangling these impacts, in which a naturally formed homoploid hybrid may be compared with its derived allopolyploid. In the recently formed polyploids reviewed here, both hybridization and genome doubling *per se* have played important roles.

This twofold effect of hybridization and chromosome doubling was first observed through studies of gene expression in the neoallopolyploid *Senecio cambrensis* (6*x*), its immediate triploid progenitor, *S. × baxteri* (3*x*), and its parental species *S. vulgaris* (4*x*) and *S. squalidus* (2*x*) (Hegarty *et al*., 2005, 2006, 2008). The greatest transcriptome shock was observed in the F_1_ triploid hybrids. This hybridization effect was ameliorated following chromosome doubling, an effect seen in wild *S. cambrensis* and in the first and subsequent generations of synthetic ‘*S. cambrensis*’ (Hegarty *et al*., 2005, 2006, 2008); after five generations, the synthetic allopolyploids displayed patterns of gene expression similar to wild *S. cambrensis*, indicating that chromosome doubling had a secondary (‘calming’) effect on changed patterns of gene expression that was detectable in the first synthetic (S_1_) generation (Hegarty *et al*., 2006, 2008). This two‐fold effect on gene expression – transcriptome shock followed by a chromosome doubling effect – has subsequently been observed in other systems, including our other focal species (e.g. *Tragopogon* (Buggs *et al*., 2011; Yoo *et al*., 2024) and *Spartina* (Chelaifa *et al*., 2010; Ferreira de Carvalho *et al*., 2017; Giraud *et al*., 2021), and *Mimulus* (Edger *et al*., 2017)).

The neoallopolyploid *Spartina anglica* is hardly distinguishable morphologically from its hybrid parent *S. × townsendii* (Huska *et al*., 2016; Wong *et al*., 2018; Granse *et al*., 2021). Transcriptome shock was observed in *Spartina* with an impact on various stress‐related genes (Chelaifa *et al*., 2010). Genome doubling *per se* induces additional expression changes, with 33% differentially expressed genes between the *Spartina* hybrid and the allopolyploid (Giraud *et al*., 2021). Notably, the number of genes mimicking the maternal (*S. alterniflora*) expression pattern and the number of *trans*gressively expressed genes increased in *S. anglica* (Giraud *et al*., 2021). Downregulation of transposable elements (TEs) was detected in hybrids, and this downregulation is accentuated following allopolyploidy (Giraud *et al*., 2021). In *Spartina*, hybridization seems to have played a more important role in methylation changes than genome doubling (Salmon *et al*., 2005). Small RNAs are also involved in this differential regulation between hybridization and polyploidy for both ra‐siRNAS (targeting repetitive sequences) and miRNA targeting genes (Cavé‐Radet *et al*., 2019, 2020). Both hybridization and genome doubling have important transcriptomic effects, but these differ in nature and intensity. Morphological and genomic changes differed between the two independently formed hybrids from the UK and France (Salmon *et al*., 2005; Giraud *et al*., 2021).

In *Tragopogon*, transcriptome shock occurs in F_1_ hybrids; major expression changes were detected following both hybridization and genome doubling (Buggs *et al*., 2011; reviewed in Soltis *et al*., 2012). Genome doubling has a major impact and is accompanied by gene silencing and homoeolog loss as well as chromosomal changes (translocations, reciprocal, and nonreciprocal aneuploidy; Buggs *et al*., 2009, 2011, 2012, 2014; Chester *et al*., 2012, 2015). The differential effects of hybridization and polyploidy are also observed on the proteome in *Tragopogon* (Koh *et al*., 2012).

Transcriptome changes were also observed in synthetic F_1_ interspecific hybrids in *Mimulus*, with higher expression levels skewed toward one of the parental species (Edger *et al*., 2017). The dominance of gene expression observed in the F_1_ interspecific hybrid further amplified in the first‐generation synthetic polyploid, reaching the greatest difference between subgenomes in the naturally occurring 140‐year‐old allopolyploid. Furthermore, the dominantly expressed subgenome has a significantly lower density of TEs near genes compared with the ‘submissive’ subgenome (Edger *et al*., 2017). Methylated TEs may decrease the expression of adjacent genes, potentially through methylation ‘spill‐over’ and/or accompanying chromatin alterations that restrict access to transcription factors (Hollister & Gaut, 2009; Woodhouse *et al*., 2014). In *Mimulus*, the remodeled methylation patterns of TEs subsequent to the initial interspecific hybridization mirrored the expression levels of nearby genes, providing additional support for the association between methylated TEs and subgenome expression dominance (Alger & Edger, 2020). This observation prompts the question of whether subgenome dominance is inherently predetermined based on subgenome differences in methylated TE density in specific hybrids or polyploid species; this aspect appears to not have been thoroughly assessed. However, in six independent resynthesized lines of *Brassica napus*, the same parental species was repeatedly observed to be dominantly expressed, mirroring the results for the *c*. 7500‐year‐old naturally established *Brassica napus* (Bird *et al*., 2021).

A recent study of synthetic *Brassica* allotetraploids challenged the expected negative association between methylated TE load and subgenome expression dominance, finding no evidence to support this relationship (Zhang *et al*., 2023). However, follow‐up studies are needed to verify the DNA methylation patterns for individual TEs using long‐read sequencing technologies (or alternative methods), which can provide a more detailed and accurate assessment of epigenetic modifications across these highly repetitive genomic regions. Bisulfite conversion, a common technique used to assess DNA methylation, coupled with the short‐read lengths generated by Illumina sequencing, can make it challenging to uniquely align reads to repetitive regions of the genome, hindering accurate methylation analysis in these regions. Importantly, other mechanisms, including *cis‐trans* regulatory conflicts, have also been proposed, which are not mutually exclusive, to explain observed subgenome expression dominance patterns (Alger & Edger, 2020). Another major remaining question is whether environmental factors play a role in determining which subgenome attains dominance in certain hybrids and polyploids. Previous studies have focused on surveying subgenome dominance in a single common environment.

- Both hybridization and genome duplication play major roles in altering gene expression, but these appear to differ in relative timing and intensity from one system to another.
- What is the impact of hybridization and subsequent genome doubling on the proteome, given that nearly all that we know about these events is based on the transcriptome?
- Is subgenome dominance inherently predetermined based on subgenome differences in TE density near genes and/or the methylation status of genes in interspecific hybrids, or is it established in the new polyploid species?
- Why is subgenome dominance not present in some new polyploids (e.g. *Tragopogon*)?

## IV. Genotypic and phenotypic diversity

### 1. Neopolyploids exhibit wide phenotypic variation intermediate between their progenitors

Many established polyploids exhibit wide variation in phenotypes intermediate between (and sometimes even transgressive to) their progenitors, and neopolyploids show a similar tendency. All neopolyploids noted here are known to have various intermediate phenotypes: floral and vegetative organs in *M. peregrinus*, *T. mirus*, *T. miscellus*, and *S. cambrensis*; and ecological niche in *Tragopogon* and *Cardamine*. Concomitantly, intraspecific phenotypic diversity may be extensive. Even though broadly intermediate in phenotype (Abbott & Lowe, 2004), the triploid hybrid, *S. × baxteri*, and the neoallopolyploid, *S. cambrensis*, tend to resemble their tetraploid parent (*S. vulgaris*) more closely in vegetative characters than they do their diploid parent (*S. squalidus*). However, the flower head (capitulum) of *S. cambrensis* is intermediate in size between *S. squalidus* (large) and *S. vulgaris* (small) (Ingram & Noltie, 1984; Abbott & Lowe, 2004; Hegarty *et al*., 2011). The larger *S. squalidus* capitulum also bears petaloid (ray) flowers around the inner disk flowers. *Senecio × baxter*i and *S. cambrensis* both bear smaller (but variable) ray flowers (Ingram & Noltie, 1984), indicating they have inherited the *Cycloidea*‐like genes that control ray flower development from *S. squalidus* (Kim *et al*., 2008; Zhang *et al*., 2017; Zhang & Elomaa, 2024). *Senecio squalidus* is outcrossing and strongly self‐incompatible, with showy ray flowers attractive to pollinators, whereas tetraploid *S. vulgaris* is an obligate selfer and does not ‘need’ to attract pollinators (Hiscock, 2000; Abbott *et al*., 2009). Studies of an introgressant hybrid form of *S. vulgaris* bearing ray flowers (*S. vulgaris* var. *hibernicus* (4*x*)) showed that it is more attractive to potential pollinators than its rayless counterpart (Marshall & Abbott, 1982, 1984a,b), and the same appears to be true for *S. cambrensis* (Hiscock and Abbott, unpublished). While triploid *S. × baxteri* is a sterile triploid, *S. cambrensis* is fully fertile and self‐compatible; this fertility and self‐compatibility appear in first‐generation synthetic allohexaploid *S. cambrensis*, indicating that the self‐incompatibility genes inherited from *S. squalidus* are not functional in wild and first‐generation synthetic allohexaploids (Hegarty *et al*., 2005, 2006; Brennan & Hiscock, 2010). This association between self‐compatibility and polyploidy appears to be widespread, with examples of self‐incompatible auto‐ and allopolyploids both having been recorded (Mable, 2004; Brennan & Hiscock, 2010).

In *Mimulus peregrinus*, both natural and synthetic polyploids show a range of phenotypes overlapping the phenotypes of the parents (*M. guttatus* and *M. luteus*; Vallejo‐Marín, 2012). Unlike most of the other recent allopolyploids, the sterile triploid intermediate is widespread and well established in the British Isles (Vallejo‐Marin & Lye, 2013). This occurrence allows for the comparison of the phenotype and performance of both naturally occurring and synthetically produced triploids. Analyses of climate suitability models suggest that the ecological niche of the natural triploid hybrids coincides with the niche of the introduced populations of its parents, suggesting that it might directly compete with them in the introduced ecological settings (Da Re *et al*., 2020).

As in other systems, there is a strong asymmetry in the formation of these triploid hybrids. Crosses in which *M. guttatus* is the maternal parent and *M. luteus* the paternal one yield viable seeds, while the opposite cross direction mostly fails (Vallejo‐Marín *et al*., 2016). This asymmetry is reflected in the parentage of naturally occurring triploids, with the vast majority having *M. guttatus* as the maternal parent (Vallejo‐Marín *et al*., 2016). Comparisons of the triploid and allopolyploid lineages show clear evidence of the classic *gigas* effect of genome doubling. Synthetic lines recreating *M. peregrinus* show increased floral and leaf organ size, larger pollen grains, guard cell size, and slower flowering time compared with its triploid versions (Meeus *et al*., 2020). Importantly, genome doubling in *M. peregrinus* brings an instant increase in sexual fertility, rescuing the sterile triploid from evolutionary oblivion. As in other recent allopolyploids, *M. peregrinus* has originated more than once (Vallejo‐Marín *et al*., 2015). However, these independent origins of *M. peregrinus* are partially interfertile (Vallejo‐Marín *et al*., 2017), demonstrating the potential of parallel evolutionary lines to merge into a single species.

The reason for this phenotypic diversity is not yet clear, but the novel gene expression patterns generated by polyploidization could be a factor, although the causal factor of novel gene expression itself could be diverse, as discussed above. Transcriptomic patterns of polyploids differ from those of their progenitors in most studied cases, resulting in novel phenotypes (Zhao *et al*., 2023), although the quantity and the pattern of change vary according to species. A spectrum of subgenome expression dominance has been reported for neoallopolyploid plants, ranging from nearly a complete lack (e.g. *Tragopogon*) to significant levels following hybridization (e.g. *Mimulus*).

At least two complementary models have been proposed to explain the spectrum of subgenome dominance observed in allopolyploids (see reviews Woodhouse *et al*., 2014; Bird *et al*., 2018; Alger & Edger, 2020). Both differences in methylated TEs near genes and genetic divergence (e.g. *cis*‐regulatory and *trans*‐regulatory mismatch conflicts) between subgenomes contributed by the diploid progenitors have been proposed as explanations that may contribute to the rapid emergence of subgenome dominance. Subgenome expression dominance in the earliest generations can ultimately lead to asymmetric evolutionary patterns between subgenomes over deeper time, including differences in fractionation (e.g. gene loss; Schnable *et al*., 2011), tandem duplication rates (Edger *et al*., 2019), selective sweeps (Hardigan *et al*., 2021), and ultimately phenotypic variation (Renny‐Byfield *et al*., 2017; Qi *et al*., 2021). Nevertheless, the balanced expression at the subgenome level does not necessarily mean a simple intermediate phenotype. The alteration of the expression pattern of a few key regulatory gene(s) could lead to phenotypic diversity or novel phenotypes. Multiple causal factors, including *trans‐*regulations between subgenomes and epigenetic regulation could contribute to phenotypic diversity, as well as spatial‐ and temporal‐specific expression differences between or among homoeologs (Eckardt, 2014; Colle *et al*., 2019; Zhao *et al*., 2020).

The novel phenotypes in allopolyploids typically appear where the progenitors have very distinct phenotypes. These phenotypes would have evolved by the distinct adaptations of the progenitors to different ecological niches, pollinators, or geographical distributions, or by genetic drift due to reduced population sizes during neopolyploid establishment. In this process, the relevant genes would have acquired different patterns of genetic and/or epigenetic regulations. At the merger of the genomes of the progenitors, these genes might be the most sensitive to the new *trans*‐regulation from the counterpart subgenomes. Despite this speculation, few studies have directly connected a novel phenotype with gene expression patterns (Zhao *et al*., 2023). As an example, in the transcriptome analysis of *Cardamine* allopolyploids that clearly differ in habitat from their progenitors, many functional genes that potentially contribute to environmental tolerance showed significant homoeolog‐specific changes in expression ratio that correspond to differences in environmental conditions (Sun *et al*., 2020; Akiyama *et al*., 2021).

- Novelty, as well as intermediacy, in morphology, physiology, and gene expression are all features of new polyploids.
- What are the underlying (epi)genetic factors that determine the phenotypic diversity of new polyploids?
- What are the parental genome features that most likely contribute to the appearance of novel traits in a polyploid derivative?

## V. Reproduction

### 1. The mating systems of new polyploids

Clonal propagation and self‐compatibility are typical characteristics of polyploid species, both of which may facilitate reproduction and avoidance of minority cytotype exclusion (Levin, 1975; Comai, 2005; Shimizu, 2022). The triploid *Cardamine insueta* indeed propagates vegetatively as a form of clonal propagation, although sexual reproduction is possible despite low fertility (Urbanska *et al*., 1997). Furthermore, one of its progenitor species, *C. rivularis*, can propagate vegetatively via ectopic meristem formation on leaves. A transcriptomic study showed that the expression of stem cell genes, including *SHOOT MERISTEMLESS*, in leaf tissues was inherited from *C. rivularis* (Sun *et al*., 2020). These data suggest that clonal propagation of *C. insueta* could have been instantaneously established at the polyploidization event through inheritance of the trait of a progenitor species, contributing to the survival of this species despite its odd ploidy.

The transition from outcrossing with self‐incompatibility to self‐fertilization is one of the most frequent evolutionary transitions in flowering plants, and it is a typical characteristic of polyploid species (Shimizu & Tsuchimatsu, 2015). Studies using synthetic and natural polyploid species, such as *Arabidopsis kamchatica* and *A. suecica* showed the dominant inheritance of self‐compatibility through epigenetic small RNA regulation (Kolesnikova *et al*., 2023; Yew *et al*., 2023), suggesting that allopolyploid plants can obtain self‐compatibility instantaneously. This is the case in the self‐compatible allohexaploid *Senecio cambrensis*, but through the intermediate sterile triploid hybrid *S. × baxteri*, which is an annual and unable to propagate clonally. Treating the branch shoots of synthetic triploid hybrids with colchicine to induce chromosome doubling generates hexaploid branches that produce fertile flower heads that produce seeds that germinate to form fully fertile, self‐compatible allohexaploids (Hegarty *et al*., 2005, 2006). However, in subsequent generations of some synthetic allohexaploid lines, a few individuals express self‐incompatible phenotypes (Brennan & Hiscock, 2010), suggesting that the self‐incompatibility haplotype inherited from the self‐incompatible diploid parent, *S. squalidus*, was not completely (epi)genetically repressed. This may reflect the different mechanisms of sporophytic self‐incompatibility operating in Asteraceae (*Senecio*) and Brassicaceae (*Cardamine*) (Allen *et al*., 2011). A summary of what is known about mating systems for each neopolyploid species highlighted in this review is provided in Supporting Information Table S1.

Lastly, the molecular basis underlying the variation in mating systems (selfing vs outcrossing) observed in the neopolyploids reviewed here remains poorly understood. However, studies in other species, such as *Arabidopsis kamchatica* and *A. suecica*, suggested that dominance regulation through small RNA can facilitate the evolution of self‐compatibility (Kolesnikova *et al*., 2023; Yew *et al*., 2023). Furthermore, it is likely that selfing confers a greater probability of survival for neopolyploids, especially annuals lacking asexual reproduction, by circumventing the challenges of finding compatible mates. This advantage may be particularly crucial for annual neopolyploids with limited time for reproduction and dispersal.

- The transition from outcrossing to self‐fertilization is a typical feature of polyploid evolution.
- What are the various mating system mechanisms operating in different polyploid systems, and are these features lineage‐dependent (i.e. at the clade levels corresponding to family or order)?
- What is the timeframe for shifts in mating systems in polyploid lineages?

## VI. Repeated evolutionary experiments

### 1. Does evolution repeat itself?

Several of the recently formed polyploids have formed multiple times in nature: *Senecio cambrensis*, *Tragopogon mirus*, *T. miscellus*, *Cardamine insueta*, and *Mimulus peregrinus*. The parents of *Spartina anglica* have hybridized twice in Europe, forming two established F_1_ hybrid populations, but only one event was followed by genome doubling. *Mimulus peregrinus* has formed twice, once in mainland Scotland and a second time in the Orkney islands, with genetic analyses showing that each origin is derived from local, sterile triploid hybrids (Vallejo‐Marin *et al*., 2015). In the *Tragopogon* allopolyploids (which have each formed over 10 times independently; Soltis *et al*., 1995; Cook & Soltis, 1999; Symonds *et al*., 2010), evolution has clearly repeated itself across multiple independent formations of the polyploids in nature and across synthetic lines, resulting in repeated changes in patterns of gene silencing and loss, gene expression, reciprocal aneuploidy, and chromosomal translocations (Buggs *et al*., 2009, 2011, 2012, 2014; Chester *et al*., 2012, 2015; Soltis *et al*., 2012).

There were two origins of allohexaploid *Senecio cambrensis* (Abbott *et al*., 1983; Ashton & Abbott, 1992; Harris & Ingram, 1992). This recent allohexaploid species provides a good example of how the movement of plants by humans can facilitate the evolution of new species via hybridization because the diploid parent of *S. cambrensis*, *S. squalidus* (Oxford ragwort), is itself a recent homoploid hybrid species originating in two gardens in the UK through the introduction of two exotic *Senecio* species, *S. aethnensis* and *S. chrysanthemifolius*, from Sicily in the late 17th century (Nevado *et al*., 2020). *Senecio squalidus* formed from a hybridization event in the garden of the Duchess of Beaufort at Badminton, Gloucestershire, where both parent plants were growing (Harris, 2002; Nevado *et al*., 2020). At the start of the 18th century, hybrids were shared with the Oxford Botanic Garden, which was already cultivating *S. chrysanthemifolius* (Harris, 2002). Backcrossing between the hybrid and *S. chrysanthemifoliu*s over the following 100 years most likely gave rise to *S. squalidus*, which then escaped from the Botanic Garden and spread across the UK via the burgeoning railway lines of the Industrial Revolution (Abbott *et al*., 2009). On its journey of rapid colonization, *S. squalidus* hybridized with the native weedy species *S. vulgaris* to create two neoallopolyploid species, *S. cambrensis* (6*x*) and *S. eboracensis* (4*x*), and a tetraploid introgressant *S. vulgaris* var. *hibernicus* (Abbott *et al*., 2009). As in *Tragopogon*, repeated patterns of evolution were observed across the natural polyploids *S. cambrensis* and *S. vulgaris* var. *hibernicus*. *S. eboracensis* (now extinct in the wild) appears to have evolved just once, in York (Lowe & Abbott, 2003). These evolutionary events can be repeated in synthetic lines of *S. cambrensis*, where transcriptome shock in triploid hybrids is followed by secondary expression changes induced by chromosome doubling, resulting in gene expression patterns resembling those seen in natural *S. cambrensis* (Hegarty *et al*., 2006, 2008). As in *Tragopogon*, these data suggest that some aspects of polyploidization at the genetic level are hard‐wired and the direct result of the combination/interaction of the parental genomes.

The multiple origins of *Tragopogon* and *Senecio* polyploids also permit examination of the complex scenarios that may occur postrecurrent polyploidization and the subsequent migration of these lineages and possible gene flow between the new polyploid lineages. The complexity of these demographics can be further enhanced by recurrent hybridization between the neopolyploid and one or both diploid parents. In *Tragopogon*, the separate polyploid formations are largely isolated geographically, but there is some evidence for gene flow between populations of independent origin and between allotetraploids and their diploid parents in several locations (Soltis & Soltis, unpublished data).

In contrast to *Tragopogon* and *Senecio*, the parents of *Spartina anglica* have independently formed F_1_ hybrids two times with the same maternal (*S. alterniflora*) and paternal (*S. maritima*) parents, but only one of these hybridizations was followed by genome doubling. In contrast to the examples above, the two hybrids display marked phenotypic differences despite their similar genetic background. Furthermore, the two F_1_ hybrids displayed > 25% differentially expressed genes and exhibited different expression patterns (Giraud *et al*., 2021).

Genetic data indicate there have been at least two independent origins of the triploid *C. insueta* (Zozomová‐Lihová *et al*., 2014). Furthermore, pentaploid and hexaploid *C. schulzii* must have originated through additional hybridization events between *C. insueta* and the autotetraploid *C. pratensis* (Mandáková *et al*., 2013). Interspecific hybridization events have thus been common in a single small village in the Swiss Alps within just the past 150 years.

- Some aspects of the evolutionary trajectory of polyploid genomes may be hardwired, repeated across multiple origins of a newly formed polyploid.
- What are the genomic and genetic factors that promote this ‘repeatability’?
- Are the outcomes of polyploidization predictable based on features of the parents?
- What are the trajectories of separate polyploid formations through time? Will they diverge in response to selective pressures?

## VII. New polyploids afford unique opportunities to distinguish inherited traits and rapid evolution

When studying established polyploid species, it is not easy to distinguish whether a new trait in the polyploid evolved instantaneously at the time of polyploidization, through rapid evolution following polyploidization, or more gradually since polyploid formation. Contemporary speciation provides a unique opportunity to observe the exact environmental differences between progenitor and polyploid species. For example, niche differentiation and the origin of clonal propagation in *C. insueta* were important key traits for the establishment of this allopolyploid species and appear to have occurred instantaneously with its formation. More generally, the traits of progenitor species can be transmitted to newly formed allopolyploids unless the traits are recessive. These observations highlight an important gap in our current observations: we are observing only established contemporary species and current phenotypic variation, but those species and traits that we observe, such as niche divergence and reproductive shifts, must have been selected as advantageous traits for the polyploid among many more unobserved polyploidization events. Larger‐scale field observations, including ‘unsuccessful’ polyploidization events, are essential for understanding both the rate of formation and the adaptation of incipient polyploid species. The studies of contemporary polyploidy provide direct support for the importance of inherited and merged traits observed in established polyploid species (Akiyama *et al*., 2021; Shimizu, 2022).

- Some traits (morphological, mating system, and habitat) are instantaneously inherited.
- Most polyploidizations fail. What are the main causal factors leading to the successful vs unsuccessful survival of newly formed polyploids?

## VIII. Changes through time – newly formed vs older polyploids

### 1. Assessing whether changes in new polyploids reflect patterns in older established polyploids

The extent to which changes observed in young polyploids reflect patterns seen in more established, older polyploid systems is unknown and understudied. Are phenomena that characterize older polyploids already present in the early generations following polyploid formation, and at what rates do such phenomena change as polyploids age? For example, if there are initial changes in cell size associated with genome doubling and possible resulting changes in organ size, how do these properties change through time? Experimental studies with natural and resynthesized polyploids have established that cell size (pollen size, guard cell size) often increases with genome doubling (Stebbins, 1971; Doyle & Coate, 2019). Changes in cell size also translate in immediate gains in organ size and in slower developmental times (Levin, 1983; Soltis *et al*., 2014). For example, in both auto‐ and allopolyploid *Mimulus*, genome doubling in natural and synthetic lineages results in polyploids with larger flowers, leaves, and stems but which take longer to flower and produce fewer flowers (Simón‐Porcar *et al*., 2017; Meeus *et al*., 2020). Whether the *gigas* effect is persistent over evolutionary time or how it might contribute to differences in the performance of polyploids (Maherali *et al*., 2009) is not well understood. Variation in fertility among newly formed polyploids has also long been of interest, with recent insights emerging from analyses of synthetic *Arabidopsis* autopolyploids (Westermann *et al*., 2024).

Efforts to approach morphological and genetic/genomic change through time have involved analysis of related natural polyploids in the same genus of greater age (Mavrodiev *et al*., 2015; Boatwright *et al*., 2021) or synthetic generations through time (e.g. Gaeta *et al*., 2007; Spoelhof *et al*., 2017; Jordon‐Thaden *et al*., 2023), but more work is needed. Polyploid genomes undergo evolutionary changes, including gene loss, leading to divergence from the initial polyploid state. Genome fractionation (loss) is evident even at the very early stages of polyploid evolution. For example, in *Tragopogon* and *Senecio*, gene loss and silencing as well as chromosomal change are present in both naturally occurring populations and the first few generations of synthetic polyploids (Hegarty *et al*., 2005, 2006, 2008; Buggs *et al*., 2009, 2012). Furthermore, these changes continue through time, as observed via analysis of older polyploids and their diploid parents (Mavrodiev *et al*., 2015; Boatwright *et al*., 2021). The rapid loss of DNA content in young polyploids signals the dynamic nature of genome evolution in young *Tragopogon* allotetraploids. Moreover, these changes through time have been reported more broadly in other plants, as well as in animals and yeast (Mayfield‐Jones *et al*., 2013).

- What is the extent to which changes in old polyploids reflect patterns in young polyploids?
- What general patterns govern the retention and loss of genetic content, as well as the role of the environment in driving patterns of genome evolution?
- Is there evidence of selection for the retention of one parental copy over the other?
- Are there biases on which parental gene copy is lost? Can these biases be predicted?

## IX. Every polyploid tells a story

### 1. Each new polyploid species has unique characteristics

Although we have stressed commonalities among the newly formed polyploids, there are many differences among each of these young species. Each new polyploid is an individual story in the early stages of evolution and therefore merits more investigation. In some of the new polyploids, there is a parental genome bias, whereas in others this is not the case (as described in the previous section). In some cases, hybridization seems to play a more significant role than genome doubling, but the relative roles differ from one recent polyploid to the next. In some cases, gene loss seems to predominate, whereas in other recent polyploids, gene silencing is more prevalent. Some new polyploids exhibit immediate chromosomal changes (e.g. structural rearrangements and chromosome content), while others show no evidence that this occurs immediately. However, there are gaps in our understanding of these properties because these neopolyploids have not been investigated in a concerted fashion. Different investigators have focused on different aspects of the polyploidy process (Table S1).

- New polyploids should be compared for the same processes using the same methods, with similar quality reference genomes, to address a consistent suite of questions, providing a foundational model study.
- What features of the parental genomes are the major drivers of change in newly formed polyploid genomes?
- Are there lineage (phylogenetic) effects, that is, do some clades have different drivers than others?
- What aspects of polyploidization are hard‐wired and determined by features in the parental genomes?

## X. Factors of formation

### 1. New polyploids often form as a result of human activities

A consistent theme in the formation of these recently formed allopolyploid species is that all have formed as a result of human intervention and disturbance (Vallejo‐Marín & Hiscock, 2016). *Spartina anglica* formed following the introduction of one parent from its native range in eastern North America to marshes in Europe (Ainouche *et al*., 2004). The story is similar, but more complex, in the formation of *Senecio cambrensis*, with one parent being a recent homoploid hybrid species that arose through hybridization between two *Senecio* species introduced to the UK from Sicily, and the other being a native tetraploid species (Abbott *et al*., 2009; Nevado *et al*., 2020).

In both allopolyploid *Mimulus peregrinus* and the two neotetraploid Tragopogons, all of the diploid parents are examples of intercontinental movement of plants by humans. In *Mimulus*, diploid parents were transported from North America to the UK as ornamentals (Vallejo‐Marín, 2012). Both *Tragopogon mirus* and *T. miscellus* formed in the western United States following the introduction of the three diploid parents from Eurasia. One diploid parent, *T. porrifolius*, was likely deliberately introduced as a food plant, and the other two (*T. dubius* and *T. pratensis*) were likely accidentally introduced, perhaps with contaminated grain (Soltis *et al*., 2023).

Most of these systems present an ethical dilemma in that the allopolyploids are native (and sometimes rare) species in their area of formation, but one or both parents are exotic: thus, do the polyploids merit protection or eradication? In contrast to the other recently formed polyploids, *Cardamine insueta* formed from species native to Switzerland, but nonetheless, because of human disturbance. The new polyploids formed in the Swiss Alps following deforestation and land use change to grazing, bringing into contact diploid progenitors with different ecological niches (Urbanska‐Worytkiewicz & Landolt, 1972; Urbanska‐Worytkiewicz & Landolt, 1974).

Given the increased tempo of human movement and migration, how many more similar scenarios will we observe in the coming decades? Indeed, how many other new polyploids have already formed via human‐mediated transport to new ranges, disturbances and land use changes but have not been detected (Vallejo‐Marín & Hiscock, 2016)? It is easy to imagine many new polyploids going undetected, and we emphasize that all the recently formed polyploids addressed in this paper were detected and described by highly observant systematists, cytogeneticists, and naturalists.

- Human disturbance creates opportunities for new polyploids to form.
- How many other new polyploids have formed that we (the botanical community) have missed?
- What are the implications of global change and increased human‐mediated disturbance – will there be more opportunities for new polyploidizations?

## XI. Major gaps: other facets of evolution to be learned from new polyploids

### 1. Current work sets the stage for future studies

Despite the intensive efforts to understand the ecological and evolutionary advantages of polyploids, there remain gaps in our knowledge. First is the measurement of key traits and environments *in natura*, or in naturally fluctuating environments (Shimizu *et al*., 2011; Kudoh, 2016). Our knowledge of the ecology and ecophysiology of the young polyploids considered here and their parents is limited. Contemporary speciation provides a unique opportunity to measure key traits and environments relevant to the speciation between progenitors and the polyploid species at the place and time of birth. For example, habitat differences accompanying the speciation of *C. insueta* support an interpretation of instantaneous niche differentiation. In addition to visible phenotypes, for example morphology and preference for a specific ecological factor, less visible factors that could contribute to the speciation of polyploids also likely exist. These factors may include the phenotypic traits affecting interactions with other species, such as pollinators, pathogens, or herbivores. In *Cardamine*, two origins of the triploid *C. insueta* and the subsequent cross with *C. pratensis* to form *C. schulzii* were reported (Mandáková *et al*., 2013; Zozomová‐Lihová *et al*., 2014). Three allopolyploidization events in the past 150 years suggest that pollination of different species is not specific, an inference that can be tested by *in situ* observation. Another critical gap is the lack of knowledge connecting the genome, epigenome, transcriptome, and phenome. We have many examples in which this connection is clear via intensive studies using model species, but for most traits, there is a lack of understanding of the genetic mechanism(s) controlling those traits, particularly in nonmodels, such as the new polyploids emphasized here. We propose that a study focusing on a phenotypic difference between a new polyploid and its progenitors could provide a good opportunity to reveal such mechanisms.

- We have learned a lot in the study of newly formed polyploids, but major gaps remain.
- What are the ecophysiological consequences of polyploidy? Such data are lacking for most new polyploids.
- Do the transcriptomic changes observed reflect the proteome? We know very little about changes at the protein level.
- What are the interactions between new polyploids and other species, such as pollinators, herbivores, fungi, and bacteria?
- Which (epi)genetic changes have contributed to the observed phenotypic changes of specific traits in neopolyploids?

## XII. Conclusions

To facilitate a comparative analysis across the species discussed, we have included Table S1, which summarizes the current state of knowledge for each species regarding the key topics covered in this review. However, we acknowledge the inherent limitations of drawing unified conclusions across such diverse model systems, given the variation in research objectives and methodologies. Nonetheless, this table provides a concise overview of key findings and highlights areas where further research is needed to address knowledge gaps and move toward a more generalized understanding of polyploid evolution.

Polyploidy has played a significant role in the evolution of life. While most polyploid species studied to date are ancient, a few model neopolyploid plant species have emerged, offering unique opportunities to study the immediate impacts of genome doubling in nature. Previous studies on diverse neopolyploids have revealed complex genetic and epigenetic changes, including altered gene dosage and expression, DNA methylation patterns, and transposon activity. Both hybridization and genome doubling contribute to these changes, but their relative impact varies among different allopolyploid species. Interspecific hybridization can induce significant transcriptome shock, while genome doubling may lead to further alterations in gene expression and epigenetic modifications. Interestingly, some polyploids exhibit subgenome expression dominance, where one parental subgenome is more highly expressed than the other subgenome(s). This dominance may be influenced by the density of certain TEs and potentially by environmental factors.

Despite significant advances in understanding polyploid evolution, many knowledge gaps remain. These include the extent of epigenetic changes in influencing subgenome expression dominance, stabilizing the newly formed nucleus following transcriptome shock, and ultimately shaping the proteome. Addressing these gaps will enhance our understanding of polyploid evolution, and its impact on observed phenotypic variation, adaptation, and speciation.

Newly formed polyploids may exhibit diverse phenotypic variation, often intermediate to their progenitors' phenotypes. This diversity can be observed in morphology, physiology, and gene expression, with some polyploids displaying novel traits. The underlying mechanisms driving this diversity are still unclear, but novel gene expression patterns and epigenetic modifications may play a role. While some polyploids (e.g. *Spartina anglica*, *Tragopogon mirus*, and *T. miscellus*) have successfully expanded their ranges and outcompeted parental species, others (e.g. *Senecio cambrensis*) face challenges in establishing and maintaining populations due to competition and extensive niche overlap with their parents. Niche differentiation is crucial for polyploid establishment and persistence. Previous studies on neopolyploids, such as *T. mirus*, *T. miscellus*, and *Cardamine insueta*, highlight the importance of both abiotic and biotic factors in shaping their ecological niches. Future studies to gain an improved understanding of the complex interplay between genotype, phenotype, and environment in neopolyploids are crucial for predicting their evolutionary trajectories and ecological impacts.

Polyploidy has not only played a significant role in the evolution of plants, contributing to the splendid diversity in nature, but has also been leveraged for agriculture over the past several thousand years. Allopolyploidization can lead to hybrid vigor, which may be expressed as larger plant or organ sizes, extended blooming periods, increased yield, and improved crop quality. Neopolyploids often exhibit enhanced tolerance to diverse environmental conditions, making them adaptable to different climates and soils. This adaptability is crucial in the face of climate change and can contribute to climate‐resilient agriculture, agricultural productivity, and sustainability. The study of naturally occurring polyploids provides a valuable template for understanding how polyploidy can be further leveraged in agriculture. However, current breeding practices often select against diversity, limiting the potential benefits of polyploidy. By adopting a more holistic approach, informed by the study of naturally formed neopolyploids, breeders can harness the full potential of polyploidy to develop resilient, high‐performing crops and ornamental plants that meet the diverse needs of industry, consumers, and the environment.

## Competing interests

None declared.

## Author contributions

PPE, DES, SY, MV‐M, RS‐I, KKS, AS, SH, MA and PSS contributed to the planning and writing of the manuscript.

## Disclaimer

The New Phytologist Foundation remains neutral with regard to jurisdictional claims in maps and in any institutional affiliations.

## Supporting information


**Table S1** Detailed information on the seven neopolyploid systems featured in this review (*Cardamine*, *Mimulus*, *Spartina*, *Tragopogon*, and *Senecio*), including their genomic characteristics and phenotypic traits.
Please note: Wiley is not responsible for the content or functionality of any Supporting Information supplied by the authors. Any queries (other than missing material) should be directed to the *New Phytologist* Central Office.
